# Flow cytometric evaluation of monoclonal antibodies for cross-reactivity with feline leukocytes

**DOI:** 10.3389/fvets.2026.1778256

**Published:** 2026-03-18

**Authors:** H. Krüger, A. Moritz, B. Roshani, S. Neumann

**Affiliations:** 1 Institute of Veterinary Medicine, Georg-August University Goettingen, Goettingen, Germany; 2 Clinic of Small Animals-Internal Medicine, Faculty of Veterinary Medicine, Justus-Liebig-University Giessen, Giessen, Germany; 3 Unit of Infection Models, German Primate Centre, Leibniz-Institute for Primate Research Goettingen, Goettingen, Germany

**Keywords:** CD markers, cross-species recognition, FACS, feline immunology, immunophenotyping

## Abstract

Monoclonal antibodies are essential tools for immunophenotyping, yet their use in feline flow cytometry is highly restricted due to the scarcity of cat-specific antibodies. Moreover, data on cross-reactivity with feline peripheral blood are limited. In this study, peripheral blood from four healthy cats was analysed using flow cytometry to assess cross-reactivity of 72 monoclonal antibodies, including 55 CD markers, 4 chemokine receptors, 7 cytokines, and 6 miscellaneous markers. Cross-reactivity was observed for 35 antibodies. Temporal effects on signal detection were evaluated using a long-term stability assessment of a staining panel comprising CD4, CD5, CD8, CD21, CD56, CD80, and Ki67. Signal detection was optimal immediately after staining and declined over time, and inter-individual variability among the cats was noted. These findings provide a validated set of cross-reactive antibodies and highlight temporal effects on signal detection in peripheral feline blood, supporting reliable flow cytometric analyses.

## 1 Introduction

The domestic cat (*Felis catus*) has emerged as the most prevalent companion animal within European households (1), underlining its increasing relevance in veterinary medicine and research. Given the domestic cat’s rising importance in veterinary practice, a closer examination of monoclonal antibodies advances—which takes an important place in human healthcare over the past decades—becomes essential.

In human biomedical research, cats have played a crucial role in advancing our understanding of neuronal networks (2), and they are considered promising animal models for future developments in auditory nerve implants (3). Additionally, they have contributed valuable insights to cancer research (4).

In feline medicine, infectious diseases remain a major concern, particularly Feline Immunodeficiency Virus (FIV) and Feline Infectious Peritonitis (FIP). To elucidate the immune mechanisms underlying these diseases, monoclonal antibodies targeting feline immune cell surface markers have become essential tools for immunophenotyping and functional immune analysis (5, 6).

Since the approval of the first monoclonal antibody (mAb) by the U.S. Food and Drug Administration in 1986 (7), the number of authorized mAb-based therapeutics has grown significantly. In 2024, the Paul-Ehrlich-Institut lists a total of 197 approved monoclonal antibodies for therapeutic application in human medicine. In contrast, veterinary medicine has not kept pace within the clinical integration of mAbs: to date, only three veterinary monoclonal antibodies have received regulatory approval, the first of which was authorized in 2020 (8). Within the EU, only one mAb is currently approved for use in feline patients, specifically for the treatment of osteoarthritis (9).

This disparity illustrates not only the considerable developmental gap between human and veterinary applications of monoclonal antibodies, but also a species bias favoring canine medicine. To facilitate future research and mitigate the costs associated with developing feline-specific antibodies, the establishment of a comprehensive repository of validated, cross-reactive mAbs suitable for use in cats could serve as a valuable tool in accelerating both preclinical studies and therapeutic innovation.

To date, only few anti-cat antibodies are commercially available and the number of anti-human mAbs with reported cross-reactivity with cats is very limited. In this study 72 differently labeled mAbs which are directed against surface and intracellular antigens were tested for cross-reactivity against whole blood cell samples of cats. The majority of tested antibodies were anti-human, while we also included two anti-non-human primates (NHP) as well as two anti-feline antibodies.

24 different fluorochrome conjugates were used, covering the violet (Brilliant Violet 421, 510, 605, 650, 711, Horizon V450, SuperNova650, Streptavidin-Brilliant-Violet 421), blue (phycoerythrin (PE), PE-Cy5, PE-Cy7, PE-CF594, peridinin chlorophyll (PerCP), PerCP-Cy5.5, PerCP-Vio, fluorescein isothiocyanate (FITC), electron coupled dye (ECD), Alexa Fluor 488), red (allophycocyanin (APC), APC-Cy7, APC Fire 810, Alexa Fluor 700, PE Fire 700) and yellow/green laser (Super Bright 600, PE-Dazzle594).

During the evaluation of cross- reactivity we noticed differences in the signal detection according to the time point of measurement upon staining/fixation. This raised the question for how long fixated feline cells provide a sufficient signal detection. To evaluate this problem, we developed a staining panel using the evaluated cross-reactive antibodies to characterize feline lymphocyte subsets, with a particular focus on B and T lymphocytes and natural killer (NK) cells as well as proliferating and activated cells. The panel included CD4 (FITC), CD5 (Streptavidin-Brilliant Violet 421), CD8 (PE), CD21 (APC), CD56 (Brilliant Violet 650), CD80 (PE-Cy7), and Ki67 (PerCP-Cy5.5). This panel was analysed over a two-week period to determine the optimal time point for measurement.

The study evaluates the cross-reactivity profiles of commercially available monoclonal antibodies with feline peripheral blood cell subsets and chemokines, thereby establishing a foundation for future immunological research. Additionally, it defines a staining panel for the characterization of lymphocyte subsets and identifies the most suitable time point for analysis.

## 2 Materials and methods

### 2.1 Animals

To evaluate the cross-reactivity of monoclonal antibodies, EDTA-anticoagulated peripheral blood samples were collected from four client-owned domestic cats (two males and two females). The female cats were 3 years old, the male cats were 2 years old, and all animals had been neutered prior to reaching 1 year of age. The cats were presented to the Small Animal Hospital of the Veterinary Institute, Georg-August University Göttingen, for a general check-up. Informed consent was obtained from the owners regarding blood examination. Clinically, all animals were considered healthy based on their medical history, physical examination, and blood analysis, which included a complete blood count (CBC) and a basic metabolic panel. As internal controls we used samples from rhesus macaques (*Macaca mulatta*) with proven cross-reactivity with anti-human antibodies. These samples were surplus material originally collected for a previously independent study.

### 2.2 Sample collection

Blood was collected from the *V. cephalica antebrachii* into EDTA tubes using a sterile, single-use needle (1 | 20 G | 0.9 mm × 40 mm). Blood sampling was conducted with an approved animal experiment application. The CBC was performed using the ProCyte Dx (IDEXX, Kornwestheim, Germany) immediately after sample collection.

### 2.3 Isolation of PBMCs

Peripheral blood mononuclear cells (PBMCs) were isolated by density gradient centrifugation. Briefly, EDTA-anticoagulated whole blood (1 mL) was layered carefully onto 1 mL Pancoll Human (PAN-Biotech GmbH, Aidenbach, Germany) and centrifuged at 800 g for 25 min. The mononuclear cell layer at the interphase was collected and transferred to a tube containing 5 mL (PBS). Cells were washed by centrifugation at 1,200 rpm for 10 min, the supernatant was discarded, and the pellet resuspended in 5 mL PBS. Cell counts were determined using a Neugebauer improved hemocytometer.

Briefly, 1 × 10^5^ PBMCs were either left unstimulated or stimulated using a cell activation cocktail containing phorbol 12-myristate 13-acetate (PMA), ionomycin (BioLegend) and Brefeldin A (BioLegend, San Diego, CA, USA) for 6 h at 37 °C. The staining with surface and intracellular antibodies was performed as described in Section 2.4.

### 2.4 Surface and intracellular staining flow cytometry

All monoclonal antibodies (mAbs) tested in this study, and their respective clones and vendors are listed in Table 1. To ensure the functionality and cross-reactivity of some of the clones control samples from rhesus macaques with reported cross-reactivity for anti-human antibodies were simultaneously proceeded. Fresh feline peripheral blood samples were processed and stained immediately after collection. Our preliminary quality control confirmed a viability of more than 99% (dead cells less than 1%, Supplementary Figure S1). Therefore, we did not include a viability dye for the measurement of fresh blood samples. Staining was performed according to manufacturer’s instructions. Briefly, mAbs were transferred into 5 mL tubes and 150 μL of the EDTA blood sample were added. CD5 (Biotin) and Streptavidin-Brillian-Violett421 were preincubated for 15 min at room temperature (RT) in the dark and then together with the other antibodies and added to the blood samples for further 30 min. To lyse residual red blood cells (RBCs) and fix leukocytes 1 mL of RBC lysis/fixation solution (BioLegend, San Diego, CA, USA) was added followed by a 15-min incubation in the dark at RT. Cells were centrifuged for 5 min at 300 g and the supernatant was discarded. Following a washing step with phosphate-buffered saline (PBS) containing 5% bovine serum albumin (BSA) cells were suspended in 150 μL PBS/5% BSA for subsequent analysis.

**Table 1:** Monoclonal antibodies tested for cross-reactivity with cats.

Molecule	Clone	Label	Source^*^	Reactivity
CD marker				
CD3	SP34-2	AF700	A	−
CD4	L200	V450	A	−
CD4	OKT4	PerCP-Cy5.5	B	−
CD4**	3-4F4	FITC	C	+
CD5**	f43	Biotin -Streptavidin BV421	D	+
**CD5**	**UCHT2**	**PE**	**B**	**+**
CD8**	fCD8	PE	C	+
CD8a	SK1	APC-Cy7	B	−
CD8a	HIT8a	APC-Cy7	B	−
CD8a	RPA-T8	APC	B	−
		PE-Cy5	B	−
		PerCP-Cy5.5	B	−
**CD10(Hit10)**	**HI10a**	**APC-Cy7**	**B**	**+**
CD11b	ICRF44	FITC	B	−
**CD11c**	**3.9**	**PE-Cy7**	**B**	**+**
CD14	M5E2	FITC	B	−
CD14	RMO52	ECD	F	−
CD14	TÜK4	PerCP-Vio700	E	+
CD16	3G8	APC-Cy7	B	−
		FITC	A	−
CD19	J3.119	PE	F	−
CD20	2H7	FITC	B	−
		PE-Cy7	B	+
CD21	B-Ly4	APC	A	+
		FITC	A	+
		PE-Cy7	A	+
CD21	HB5	SB600	G	−
CD25	M-A251	PE-Cy7	B	−
CD27	M-T271	APC	A	−
CD27	O323	BV650	B	−
CD28	CD28.2	FITC	A	−
CD34	561	BV421	B	+
CD38	LS198-4-3	Snv605	F	+
CD45***	D058-1283	PE	A	−
		PerCP	A	**−**
CD45RA	2H4	ECD	F	−
**CD56**	**NCAM16.2**	**BV650**	**A**	**+**
CD69	FN50	BV510	B	+
		PE-Dazzle594	B	+
CD80	L307.4	PE	A	−
**CD80**	**2D10**	**PE-Cy7**	**B**	**+**
		PE-Dazzle594	B	+
CD86	IT2.2	AF488	B	+
		APC	B	+
CD95	DX2	PE-Cy7	B	+
CD117	104D2	PE	B	+
CD123	6H6	PE	B	+
CD123	7G3	PerCP-Cy5.5	A	−
**CD134**	**L106**	**PE**	**A**	**+**
CD159a	Z199	APC	F	−
CD163	GHI/61	BV711	B	+
CD279 (PD-1)	EH12.2H7	PE-Cy7	B	−
Chemokine receptors				
**CD184 (CXCR4)**	**12G5**	**PE-CF594**	**A**	**+**
CD185 (CXCR5)	MU5UBEE	APC	G	−
CD196 (CCR6)	G034E3	PE-Dazzle594	B	−
CD197 (CCR7)	G043H7	BV421	B	−
		PE-Cy7	B	−
Cytokines				
IFN-γ	4S.B3	PerCP-Cy5.5	B	−
IL-1a	364-3B3-14	PE	B	−
IL-2	MQ1-17H12	BV650	A	+
IL-4	8D4-8	APC	A	+
IL-17A	eBio64CAP17	PE	G	−
IL-21	3A3-N2	APC	B	+
TNF-∝	Mab11	BV711	B	+
		PE-Cy7	B	+
Miscellaneous				
Granzyme B	GB11	PE-CF594	A	−
**HLA-DR**	**L243**	**APC-Cy7**	**B**	**+ (weak)**
		APC-Fire810	B	+ (weak)
Ki67	B56	PerCP-Cy5.5	A	+
Ki67	Ki67	BV605	B	−
Perforin	Pf-344	FITC	H	−
γδ TCR	B1	PE/Fire700	B	−

*A—BD, B—BioLegend, C—Invitrogen, D—Southern Biotech, E—Mitenyi, F—Beckmann Coulter, G—ebioscience, H—Mabtech; +, cross-reactive; −, no positive staining; ** anti-feline, *** anti-NHP. For Clones in bold cross reactivity was not previously reported.

For intracellular cytokine staining, PBMCs were stained with surface markers and/or a live/dead stain (Zombie-Yellow, BioLegend, San Diego, CA) and fixed using RBC lysis/fixation solution. Cells were then washed twice by adding 150 μL of Intracellular Staining Permeabilization Wash Buffer (BioLegend, San Diego, CA, USA), followed by centrifugation at 1,200 rpm for 5 min, discarding the supernatant after each step. Subsequently, the intracellular antibody mix was prepared in 50 μL of Wash Buffer and added to the cell pellet, followed by incubation for 20 min at room temperature (RT) in the dark. Finally, cells were washed once more with 150 μL of Wash Buffer, centrifuged for 5 min at 1,200 rpm, the supernatant was discarded, and the cells were resuspended in 150 μL PBS/5% BSA for analysis.

**Figure 1:**
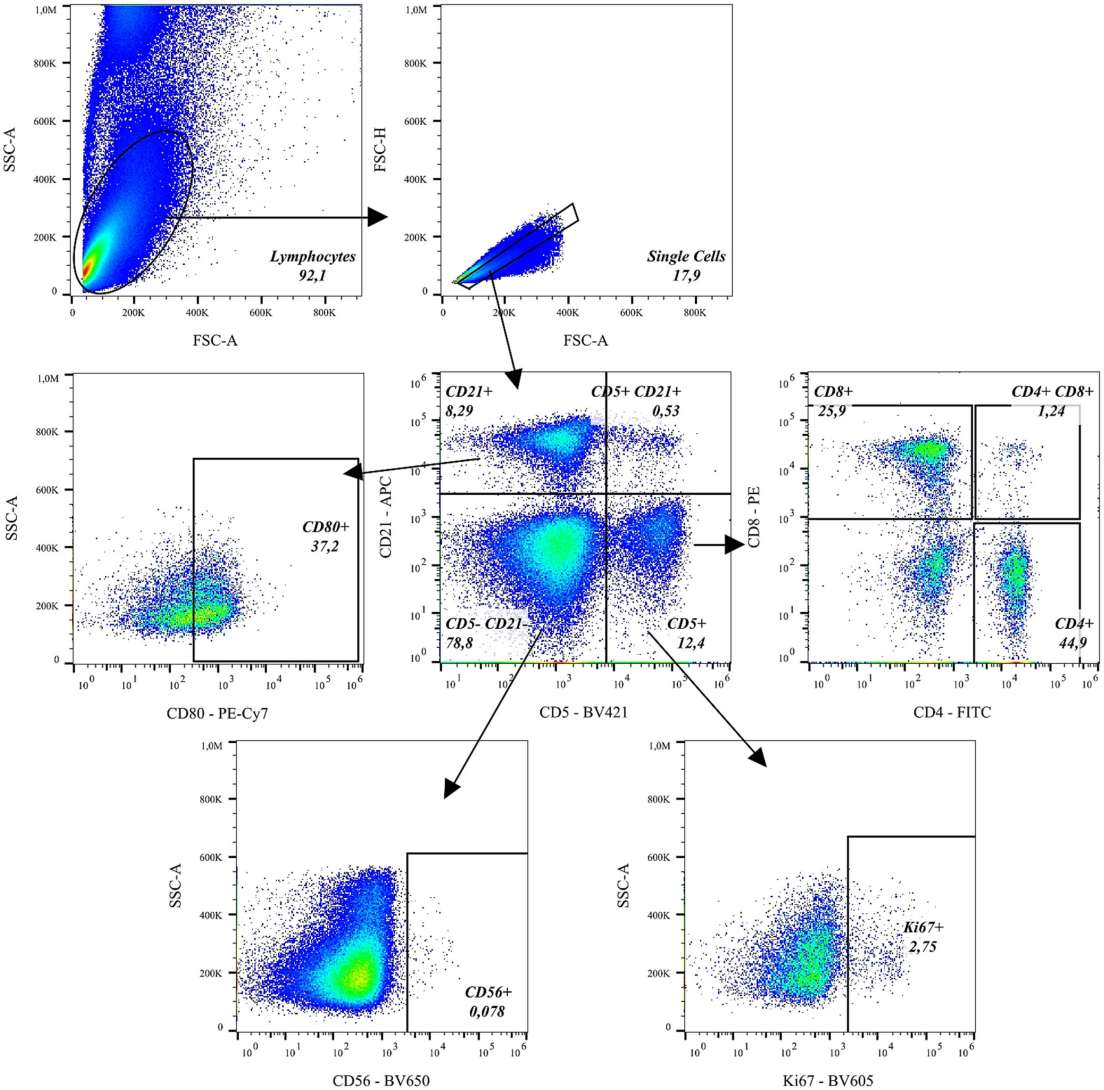
Gating strategy for feline lymphocytes. Lymphocytes were first identified based on forward and side scatter properties. Singlets were subsequently selected to exclude doublets. CD5 and CD21 expression were then analyzed to define major lymphocyte subpopulations. CD5^−^CD21^−^ cells were further gated for CD56 to identify natural killer (NK) cells. NK cells and CD21^+^ B lymphocytes were analyzed for Ki67 expression to assess proliferation. CD21^+^ B lymphocytes were further analyzed for CD80 expression. In addition, CD5^+^CD21^+^ B cells and T lymphocytes were evaluated for Ki67 expression. Within the CD5^+^ population, CD4^+^ T helper cells and CD8^+^ cytotoxic T cells were identified and subsequently analyzed for Ki67.

Cells were acquired using the ID7000™ spectral analyzer (Sony Biotechnology, San Jose, CA, USA). Unmixing was calculated using single-stained Ultra Comp eBeads (BioLegend, San Diego, CA, USA). Analysis was performed using FlowJo 10.10.0 (Treestar, Ashland, OR USA). Flow cytometric analysis was performed using a predefined gating strategy. As initial step, lymphocytes were identified based on their characteristic forward scatter (FSC-A) and side scatter (SSC-A) properties. Within the lymphocyte gate, doublets and cell debris were excluded by gating on FSC-A versus FSC-H, thereby restricting the analysis to singlet cells. Marker expression was subsequently analysed within the singlet lymphocyte population using fluorescence—based gating (see Figure 1). Supplementary Figure S1A shows representative viability data. For intracellular cytokine staining of feline and rhesus PBMCs, zombie yellow was included to exclude non-viable cells prior to marker analysis (Supplementary Figure S1B).

### 2.5 Assessment of long-term stability

Whole blood from two individuals of the same age, health status and body condition were withdrawn on the same day and simultaneously stained with FITC (CD4), BV421(CD5), PE (CD8), APC (CD21), BV560 (CD56), PE-Cy7 (CD80), PerCP-Cy5.5(Ki67) as previously described. All antibodies were titrated prior to use to determine the optimal staining concentration yielding maximal signal-to-noise ratio. Samples were fixed and stored at 4 °C in the dark until measurement up to 2 weeks and measured in a two-day interval.

## 3 Results

The results of the cross-reactivity analysis are summarized in Table 1. The tested monoclonal antibodies (mAbs) were grouped into four categories: CD markers, chemokine receptors, cytokines, and miscellaneous markers, including Granzyme B and Perforin. Antibodies exhibiting cross-reactivity (+) were distinguished from those with no detectable signal (−). For HLA-DR the detection signal was weak. Cytokines and cytotoxic entities were analysed performing an intracellular cytokine staining (ICS) in combination with stimulation through PMA/Ionomycin.

Among the 72 mAbs tested, cross-reactivity was observed for 26 CD markers (36%), one chemokine receptor, five cytokines, and three miscellaneous markers.

To our knowledge, cross-reactivity for 9 of these positively tested clones (marked bold in Table 1) has not yet been described for feline peripheral blood therefore this study provides the first description of cross-reactivity with the feline species.

**Figure 2:**
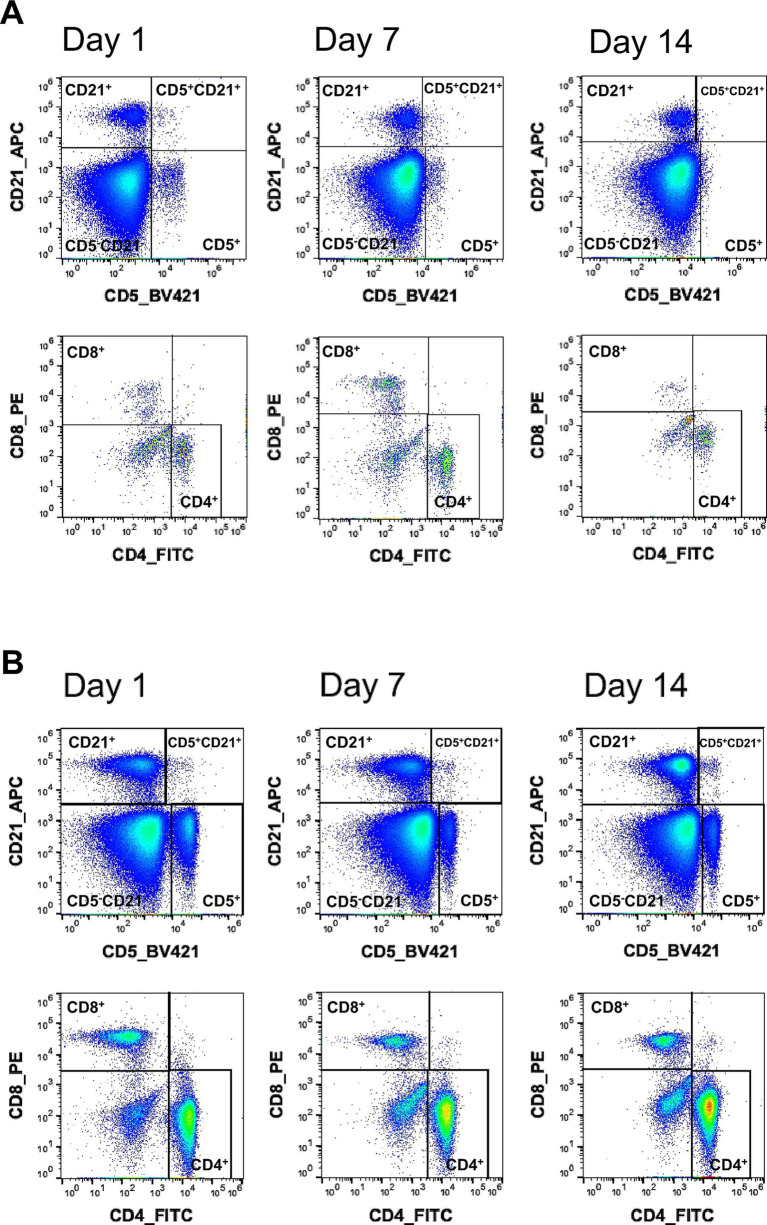
Differences in fluorescence signal quality for measurement of feline samples at different time points post staining. Peripheral blood lymphocyte populations were analyzed in two healthy cats of the same age at three time points (day 1, 7, and 14). **(A,B)** Upper dot plots show the identification of B and T lymphocytes by gating CD21 against CD5. Lower dot plots show the analysis of T lymphocyte subsets, with CD4^+^ and CD8^+^ T cells quantified in cat A **(A)** and cat B **(B)** at indicated time points.

During preliminary testing in a total of 10 cats, we observed some special features for feline blood cells. As reported previously (10), we observed smaller feline peripheral lymphocytes compared to their human or non-human primate counterparts. Furthermore, differences in fluorescence signal intensity between different cats became obvious. These differences were unrelated to age, sex or metabolic parameters, but rather may have depend on the time point at which they were measured after fixation despite storage at 4 °C and in the dark. Therefore, we evaluated a staining panel containing well-established antibodies and clones for the detection of feline B and T lymphocytes (Table 2): CD4 (3-4F4), CD5 (f43), CD8 (fCD8), and CD21 (B-Ly4). To identify NK cells and antigen-presenting cells (APCs), we included CD56 (NCAM16.2) and CD80 (2D10), both of which demonstrated cross-reactivity in our study. In addition, the cross-reactive proliferation marker Ki67 (B56) was incorporated. Figure 1 depicts the flow cytometric analysis. The gating strategy is described in detail in the Material and methods section (Section 2.4). T and B cells were distinguished based on CD5 versus CD21 expression. Surprisingly, we detected a CD5 CD21 double-positive population. Fluorescence Minus One (FMO) controls for CD21 demonstrated that the fluorescence spread from BV421 and BV650 was negligible and that this CD5^+^ CD21^+^ population sat clearly outside the defined background indicating a true biological population (Supplementary Figure S2). From the CD5^+^ population, further gating was applied to identify CD4^+^ T helper cells and CD8^+^ cytotoxic T cells. Each of these subsets (CD5^+^, CD4^+^, and CD8^+^) was then analysed for Ki67 expression to assess cellular proliferation. Within the CD5^+^CD21^+^ B-cell subset, additional gating was performed to evaluate the expression of CD80, a costimulatory molecule, and Ki67The same markers (CD80 and Ki67) were also assessed in the CD5^−^CD21^+^ B-cell population, representing a more conventional or mature B-cell phenotype. Cells negative for both CD5 and CD21 (CD5^−^CD21^−^) were gated for CD56 expression to identify natural killer (NK) cells, which were subsequently analysed for Ki67 to determine their proliferative status (FMO controls for CD80, Ki67 and CD56 are depicted in Supplementary Figure S3). This staining panel was then used to evaluate the optimal time point for sample acquisition after fixation. Blood from two cats was stained on day one and either directly measured or stored in the dark at 4 °C and measured on days seven and 14 after staining and fixation (Figure 2).

**Table 2:** Staining panel to evaluate feline lymphocytes.

Molecule	Clone	Label	Function
CD4	3-4F3	FITC	Co-receptor on helper T-cells
CD5	f43	Streptavidin + BV421	Modulates B and T cell receptor signalling thresholds
CD8	fCD8	PE	Co-receptor on cytotoxic T-cells
CD21	B-Ly4	APC	Enhances B cell activation
CD56	NCAM16.2	BV560	Contributes to NK cell cytotoxic function
CD80	2D10	PE-Cy7	Costimulatory molecule, expressed on antigen-presenting cells
Ki67	Ki67	BV605	Marker for cell proliferation

Our results indicate that the time point of measurement influences fluorescence signal detection. Figure 2 illustrates representative examples comparing the signals of CD21 versus CD5 and CD8 versus CD4 on days 1, 7, and 14. While the lymphocyte subpopulations were clearly distinguishable on day 1, the signal intensity decreased over time. These alterations were most prominent in cat A (Figure 2A) with a clear separation of the different populations on day 1, while the fluorescence signal gradually diminished over the subsequent time points. A similar trend was observed in cat B (Figure 2B); however, the decrease in signal was less pronounced. Interestingly, inter-individual variability was evident despite using samples from healthy animals of the same breed and age group and performing staining, storage, and analysis under identical conditions.

## 4 Discussion

This study provides a comprehensive overview of commercially available antibodies—both surface and intracellular markers—and their cross-reactivity with peripheral feline blood cells, which fills a gap in the characterization of feline immunophenotyping reagents. Particularly noteworthy are the surface markers CD34 and CD117, which serve as hematopoietic cell markers and to our knowledge, no prior studies have demonstrated their cross-reactivity in cats (11). As this study was designed to investigate antibody cross-reactivity in feline peripheral blood, its scope is necessarily limited to this sample type. Peripheral blood sampling was deliberately chosen to comply with ethical requirements permitting only minimally invasive procedures in clinically healthy cats. Consequently, tissue-based biological positive controls were beyond the scope of the present study. To extend the applicability of these findings and to conclusively confirm antibody specificity across hematopoietic lineages, future investigations should include samples from lymphoid organs and bone marrow.

For peripheral blood analysis, the availability of feline-reactive monoclonal antibodies (mAbs) specific for B and T lymphocytes remains limited. For B-cell detection, CD19 would theoretically provide a highly specific marker, as it is well established in human immunology (12, 13). However, cross-reactivity of CD19 with feline cells has not yet been reported, although a recent study shows promising progress toward developing a feline-specific CD19 mAb (14). In veterinary research, CD21 is commonly used for B-cell identification, but its expression is absent on early B-cell precursors (15), and either absent or downregulated on plasma cells (16) as well as early transitional B cells (17). As the CD21 clone Ly-4 hat not been extensively validated in feline samples in direct comparison with established clones such as CA2.1D6, clone-specific binding characteristics cannot be fully excluded.

The CD21 clone Ly-4 was selected for the long-term assessment, because it demonstrated clear cross-reactivity with feline lymphocytes within the antibody cross-reactivity testing performed in this study and has previously been shown to cross-react with CD21 in other mammalian species (18). Future studies directly comparing Ly-4 with the established CA2.1D6 clone will be required to further validate Ly-4 as a reliable cross-reactive reagent for feline samples.

In current literature there were so far no hints for the existence of a defined CD5^+^CD21^+^ B-cell subset in cats (19). For dogs, CD5^+^CD21^+^ cells were previously described as neoplastic B-cells (20) as well as in reactive non-neoplastic lymph nodes (21). Further investigations are needed to characterize CD5^+^CD21^+^ lymphocyte subsets in cats, as their lineage and biological relevance remain currently undefined.

For T-cell detection, CD3 serves as a reliable pan–T-cell marker in human and primate research. In veterinary medicine, however, evidence for cross-reactivity remains limited and has thus far only been demonstrated in feline lymph nodes (19) and cerebrospinal fluid (22). The CD3 clone SP34-2 did not show detectable reactivity in feline samples and could therefore not be used as a pan-T cell marker in the final panel. As a consequence, T-cell identification relied primarily on CD5 expression, which may represent a limitation in neoplastic conditions where CD5 can be downregulated. Future studies should incorporate validated feline CD3 clones, such as CD3-12, to provide more robust T-cell identification. Preliminary tests with peripheral blood samples from 10 cats indicated variations in signal detection depending on the time point of measurement upon fixation and storage. Fluorescently labelled antibodies inherently differ in the number of fluorophores attached per antibody molecule—known as the degree of labelling (DOL)—which varies between manufacturers. Both excessively high and very low DOL can negatively affect antibody performance. A high DOL may impair antigen binding and lead to reduced fluorescence due to self-quenching (23, 24), while a very low DOL may yield insufficient signal intensity (25). Furthermore, antibodies conjugated via N-hydroxysuccinimide (NHS) ester chemistry often exhibit broad and inconsistent DOL distributions, which can further decrease signal reliability (25). In the present study, no NHS-conjugated mAbs were used. We therefore conducted a long-term stability assessment as an exploratory analysis with blood samples of two cats that were processed simultaneously and measured between day 0 and day 14 post staining and fixation. While the number of animals included was limited, the data indicate a time-dependent change in fluorescence intensity during prolonged sample storage, suggesting that delayed analysis may affect signal strength under the applied conditions. Future studies including lager cohorts will be required to confirm these observations and to define standardized pre-analytical handling conditions.

Lymphocyte populations were identified based on FSC/SSC characteristics, which showed consistent and reproducible scatter profiles across samples. For expanded applications, incorporation of additional leucocyte markers, such as CD45 or CD18, would further refine leucocyte identification and enhance gating precision, particularly in more heterogeneous sample types.

## 5 Conclusion

In conclusion, this study provides a list of 35 cross-reactive antibodies for feline peripheral blood cells that can be used to address various scientific questions in feline biomedical research. Furthermore, the study identifies temporal effects on fluorescence signal detection, indicating the optimal measurement time for stained feline blood samples. In line with the principles of the 3Rs (Refine, Replace, Reduce), this study also highlights the diagnostic and scientific potential of whole-blood samples, which can be collected without sedation and within a short time frame, thereby minimizing stress for the animals and facilitating ethically responsible experimental design.

## Data Availability

The original contributions presented in the study are included in the article/Supplementary material, further inquiries can be directed to the corresponding author.

## Supplementary material

SUPPLEMENTARY FIGURE S1 Discrimination of live/dead cells and intracellular cytokine staining in feline PBMCs compared to rhesus PBMCs. **(A)** Fresh feline peripheral blood cells were first gated on lymphocytes based on FSC-A versus SSC-A, followed by singlet selection using FSC-H versus FSC-A. Live/dead discrimination was then performed using zombie yellow versus FSC-H. **(B)** Feline (top row) and rhesus (bottom row) PBMCs were gated on lymphocytes based on FSC-A versus SSC-A, followed by a singlet gate and exclusion of dead cells. PBMCs were either stimulated with PMA/Ionomycin or left unstimulated and TNF-α expression was assessed.

SUPPLEMENTARY FIGURE S2 FMO control for CD80 and corresponding full-stain in feline peripheral blood. Top row: FMO (top row) or full-stain (bottom stain) for CD80-Pe-Cy7, Ki67-Per-CP Cy 5.5 and CD56-BV650.

SUPPLEMENTARY FIGURE S3 FMO control for CD21 and corresponding full-stain in feline peripheral blood. Depicted are FMO (top row) or full-stain (Bottom-row CD21-APC versus CD5-BV421, CD56-BV650 and Ki67-Per-CP Cy 5.5.
