# Supplementary figures and images for: Flow cytometric evaluation of monoclonal antibodies for cross-reactivity with feline leukocytes
Source: Front Vet Sci. 2026 Mar 18;13:1778256. doi: 10.3389/fvets.2026.1778256 (PMC13041543; doi:10.3389/fvets.2026.1778256)

# A fresh blood cells

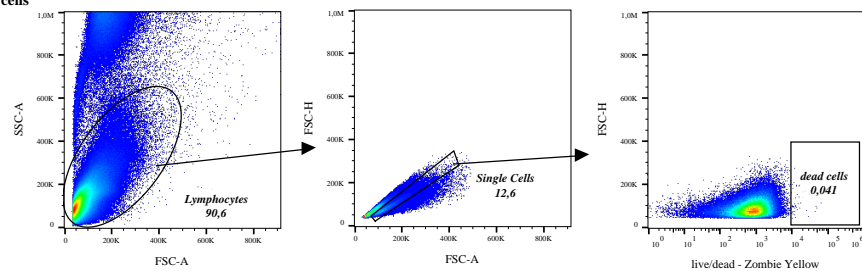

# B ICS and control with rhesus PBMCs

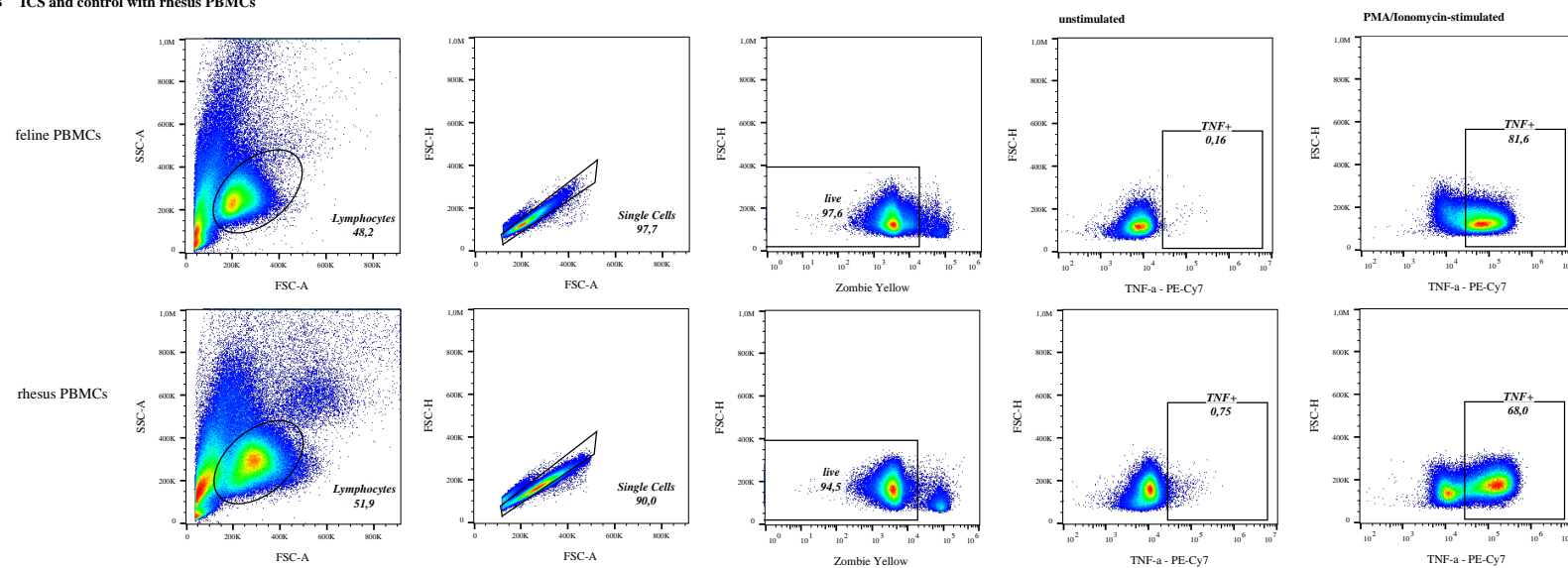

Supplement: SUPPLEMENTARY FIGURE S1 — Discrimination of live/dead cells and intracellular cytokine staining in feline PBMCs compared to rhesus PBMCs. (A) Fresh feline peripheral blood cells were first gated on lymphocytes based on FSC-A versus SSC-A, followed by singlet selection using FSC-H versus FSC-A. Live/dead discrimination was then performed using zombie yellow versus FSC-H. (B) Feline (top row) and rhesus (bottom row) PBMCs were gated on lymphocytes based on FSC-A versus SSC-A, followed by a singlet gate and exclusion of dead cells. PBMCs were either stimulated with PMA/Ionomycin or left unstimulated and TNF-α expression was assessed. [file Image_1.pdf]

**FMO**

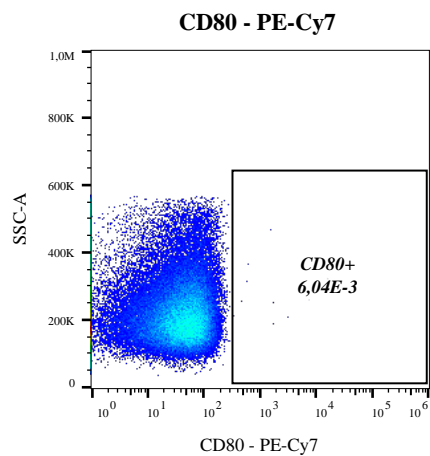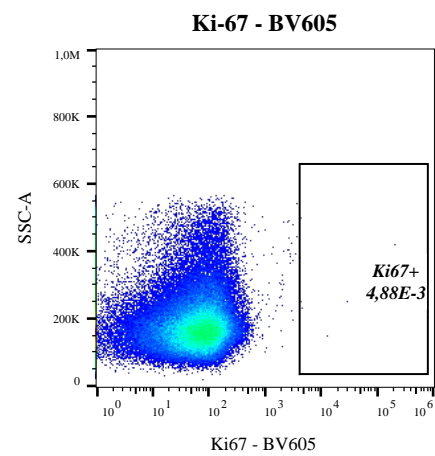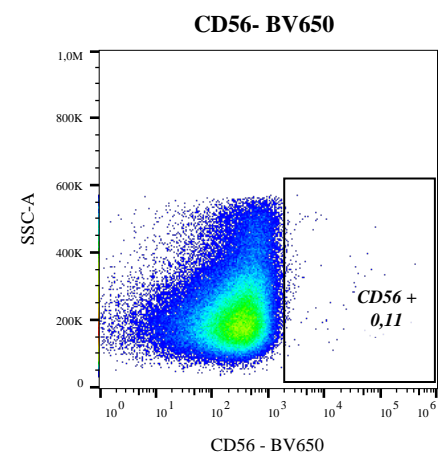

**Full stain**

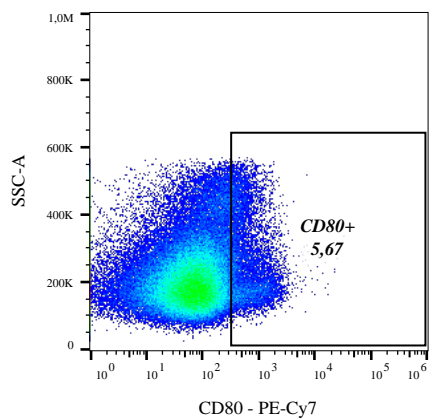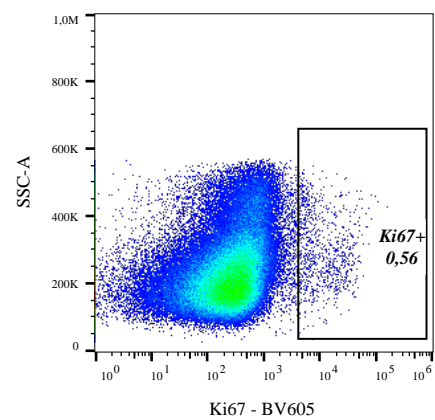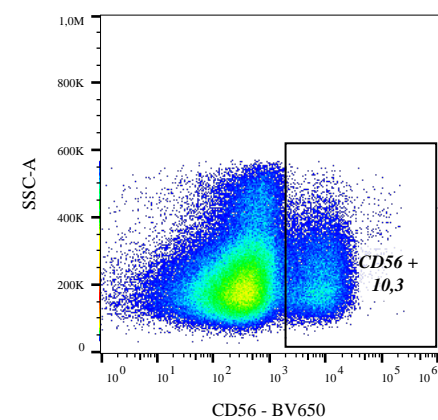

Supplement: SUPPLEMENTARY FIGURE S2 — FMO control for CD80 and corresponding full-stain in feline peripheral blood. Top row: FMO (top row) or full-stain (bottom stain) for CD80-Pe-Cy7, Ki67-Per-CP Cy 5.5 and CD56-BV650. [file Image_2.pdf]

FMO CD21 - APC

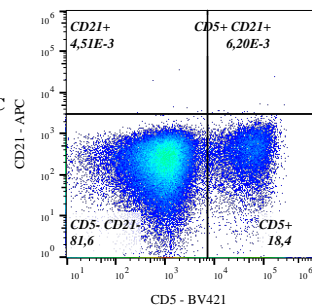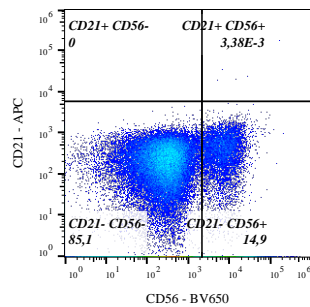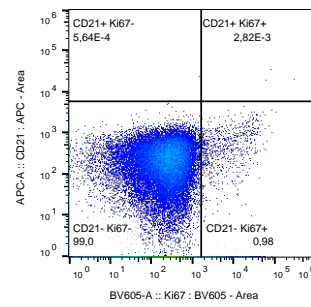

Full stain

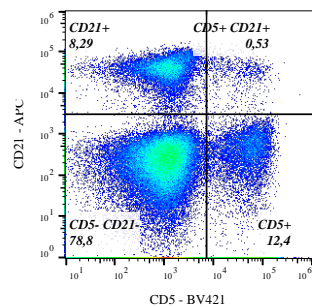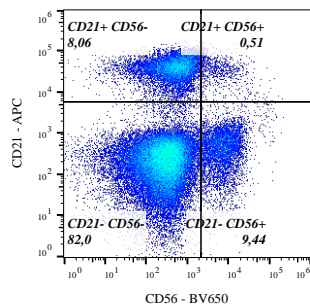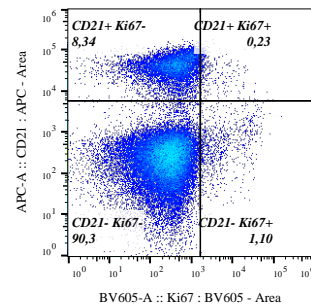

Supplement: SUPPLEMENTARY FIGURE S3 — FMO control for CD21 and corresponding full-stain in feline peripheral blood. Depicted are FMO (top row) or full-stain (Bottom-row CD21-APC versus CD5-BV421, CD56-BV650 and Ki67-Per-CP Cy 5.5. [file Image_3.pdf]
